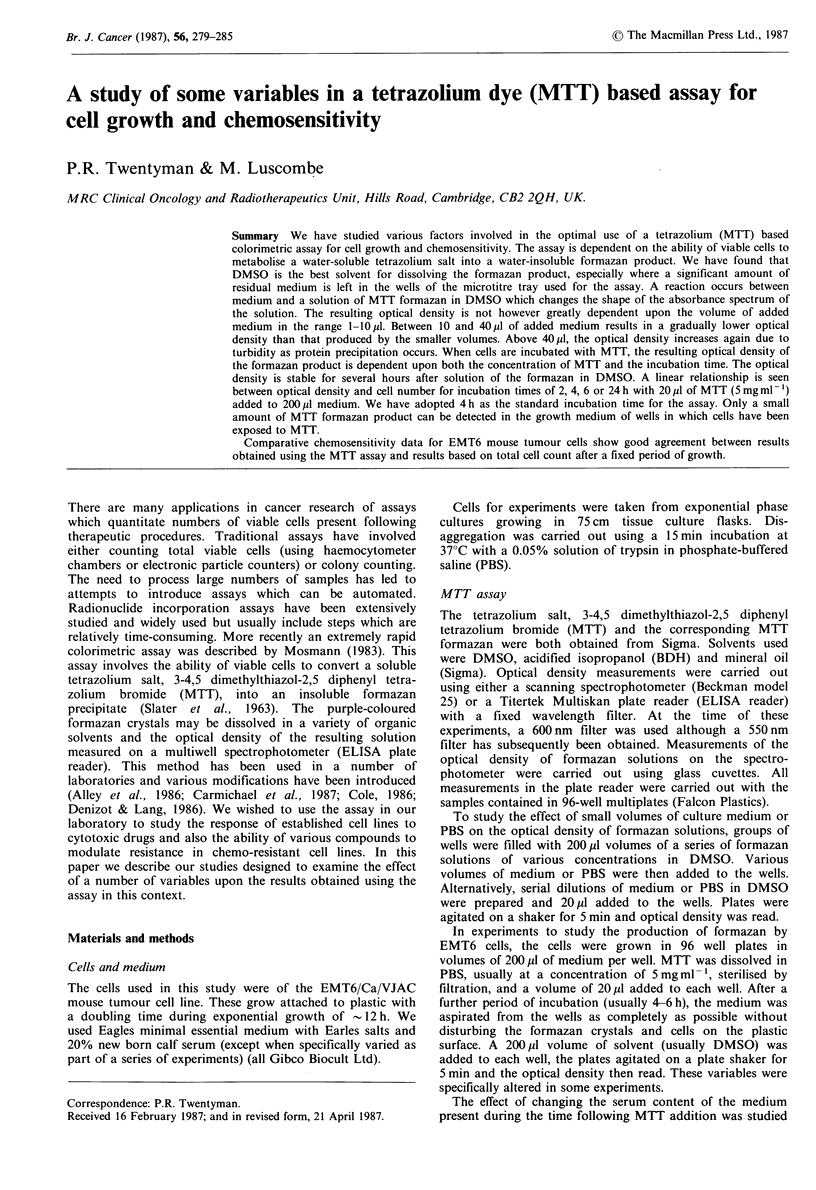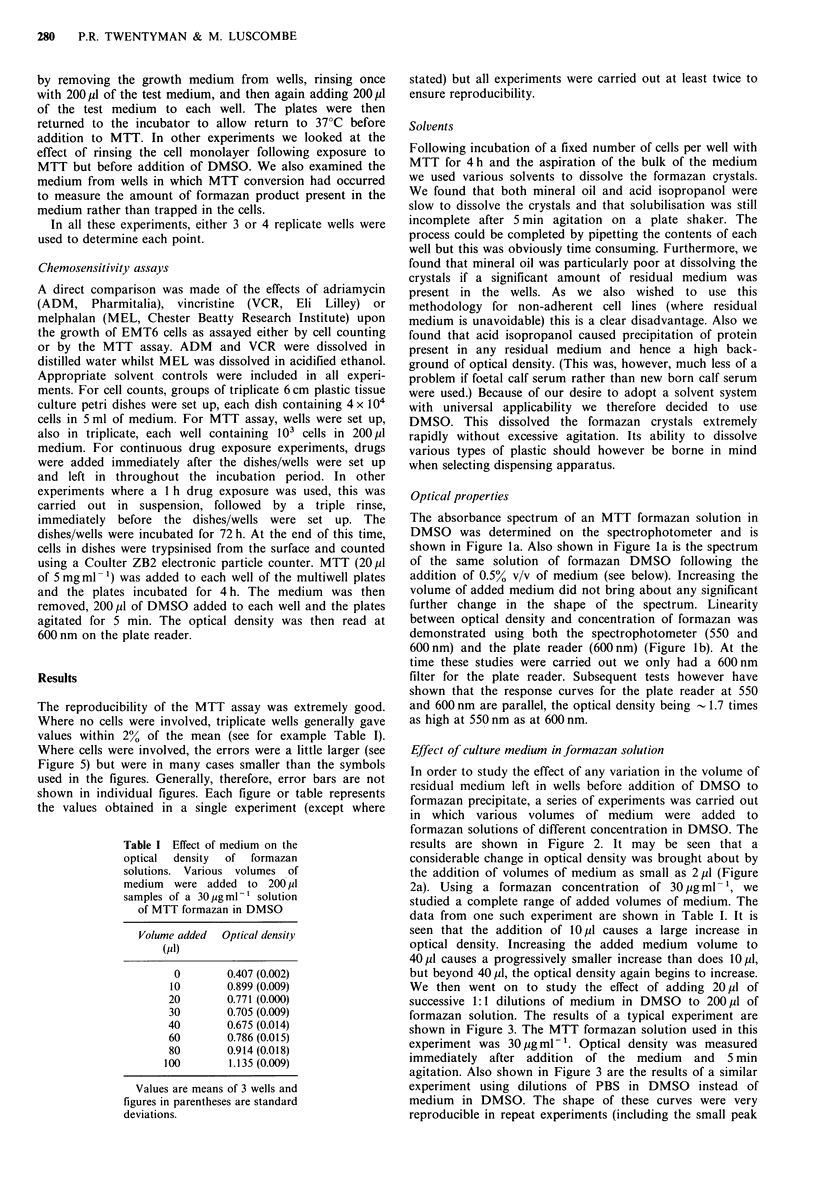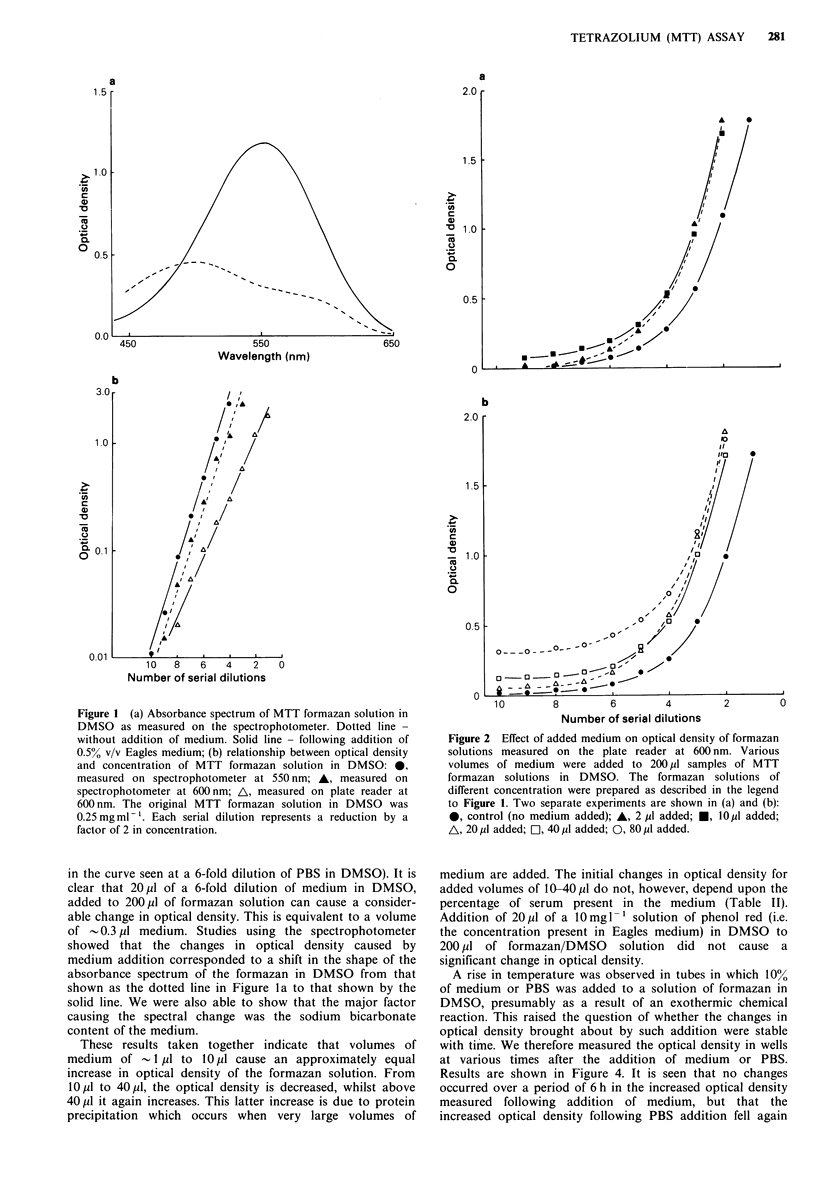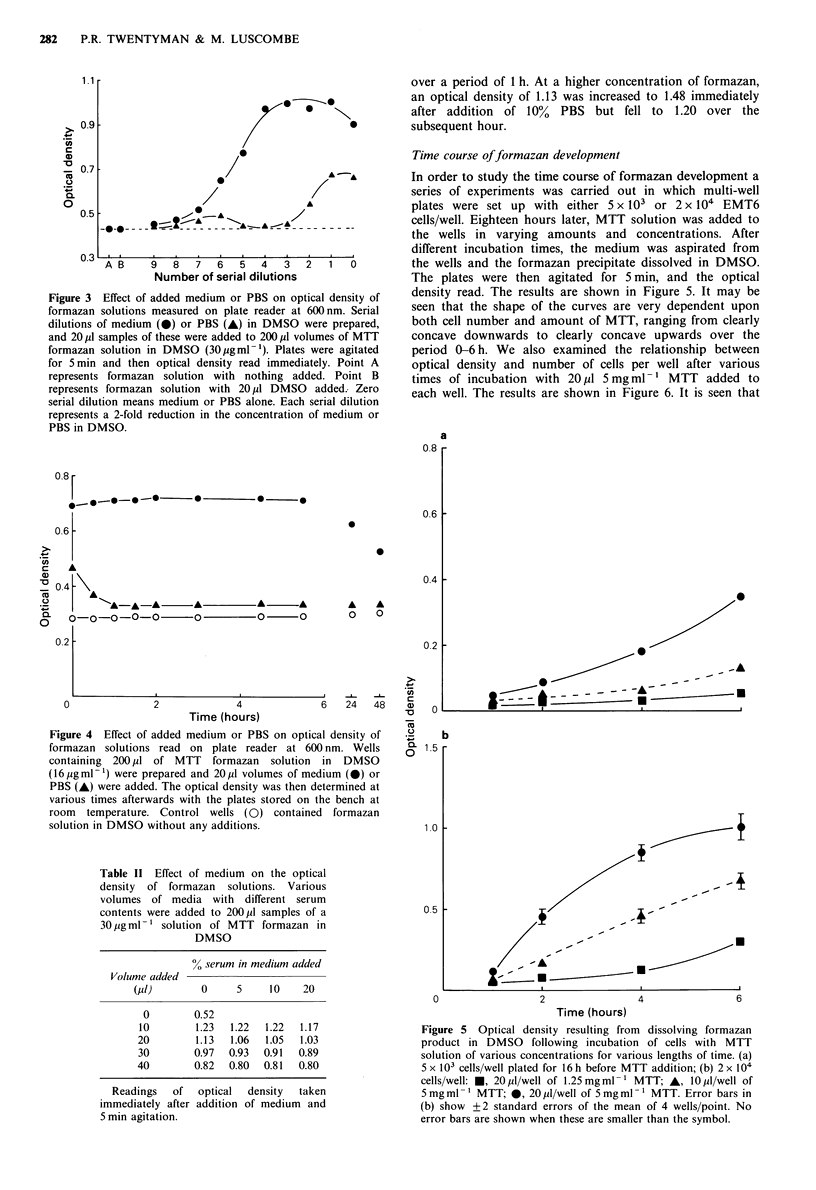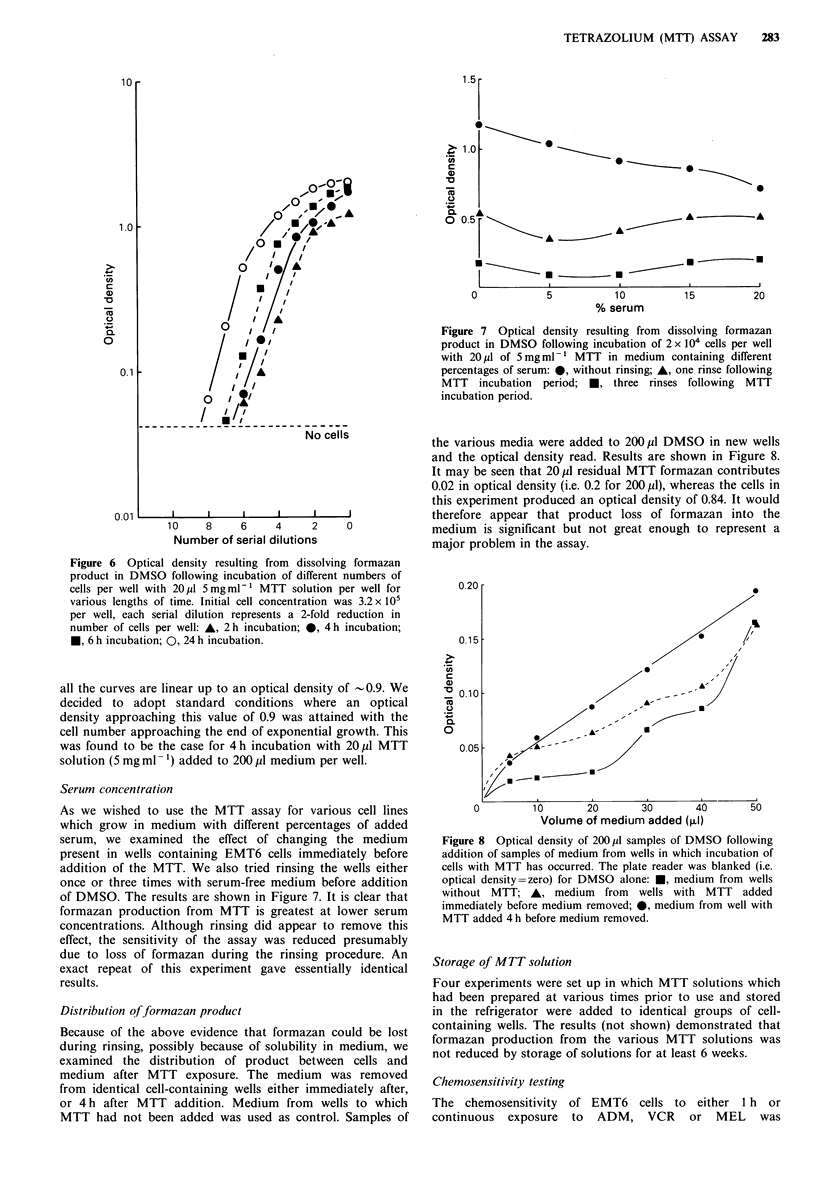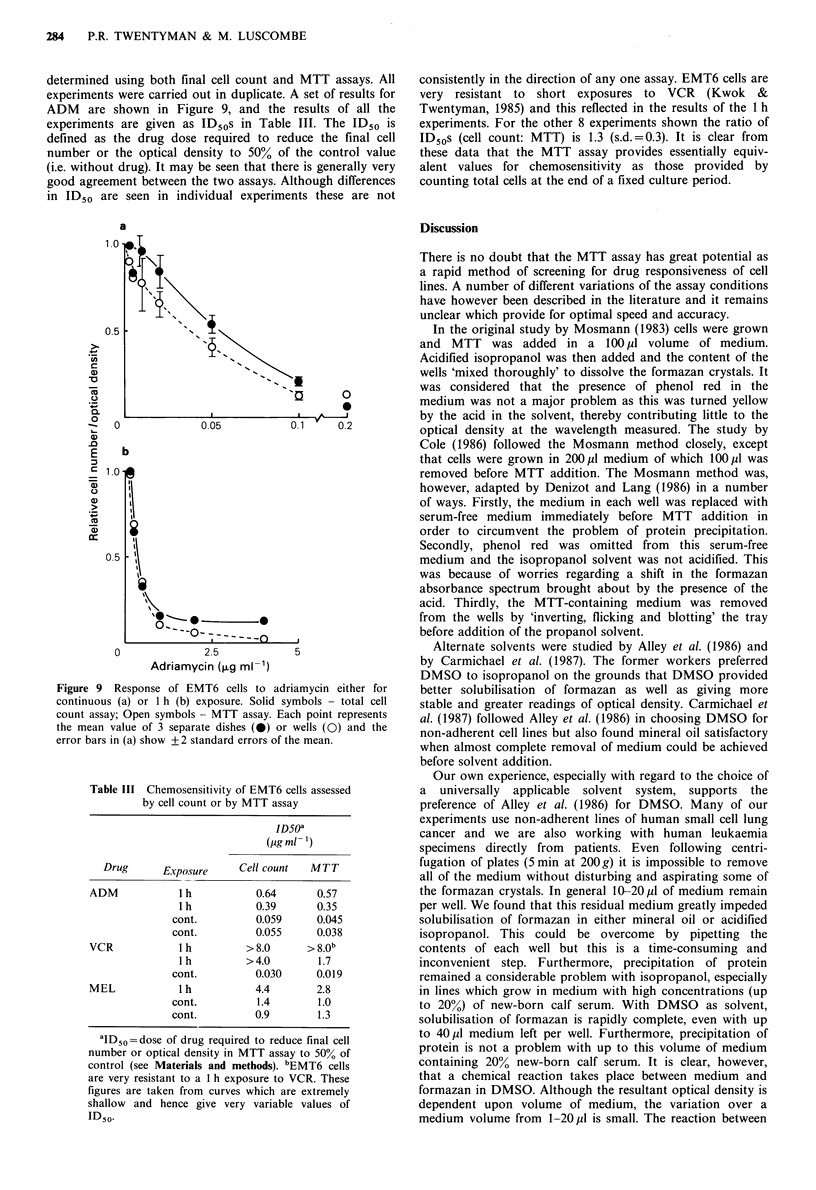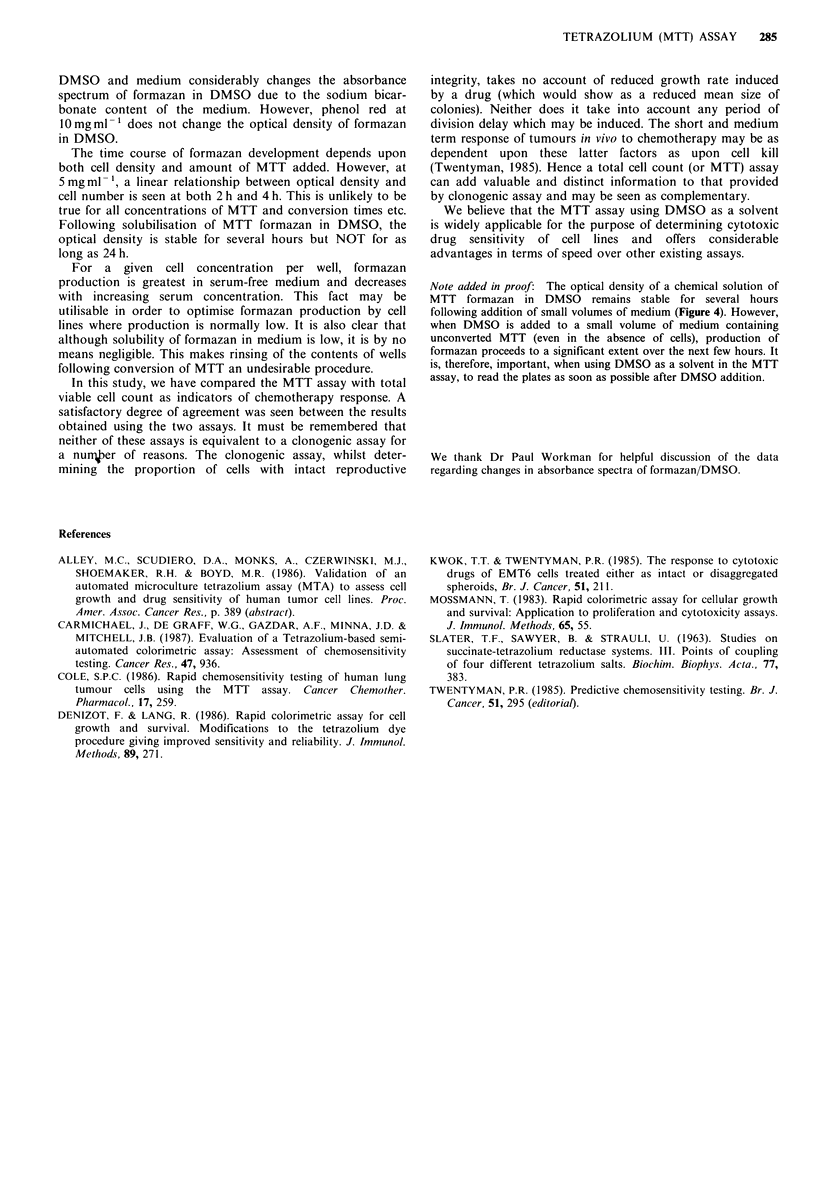# A study of some variables in a tetrazolium dye (MTT) based assay for cell growth and chemosensitivity.

**DOI:** 10.1038/bjc.1987.190

**Published:** 1987-09

**Authors:** P. R. Twentyman, M. Luscombe

**Affiliations:** MRC Clinical Oncology and Radiotherapeutics Unit, Cambridge, UK.

## Abstract

We have studied various factors involved in the optimal use of a tetrazolium (MTT) based colorimetric assay for cell growth and chemosensitivity. The assay is dependent on the ability of viable cells to metabolise a water-soluble tetrazolium salt into a water-insoluble formazan product. We have found that DMSO is the best solvent for dissolving the formazan product, especially where a significant amount of residual medium is left in the wells of the microtitre tray used for the assay. A reaction occurs between medium and a solution of MTT formazan in DMSO which changes the shape of the absorbance spectrum of the solution. The resulting optical density is not however greatly dependent upon the volume of added medium in the range 1-10 microliters. Between 10 and 40 microliters of added medium results in a gradually lower optical density than that produced by the smaller volumes. Above 40 microliters, the optical density increases again due to turbidity as protein precipitation occurs. When cells are incubated with MTT, the resulting optical density of the formazan product is dependent upon both the concentration of MTT and the incubation time. The optical density is stable for several hours after solution of the formazan in DMSO. A linear relationship is seen between optical density and cell number for incubation times of 2, 4, 6 or 24 h with 20 microliters of MTT (5 mg ml-1) added to 200 microliters medium. We have adopted 4 h as the standard incubation time for the assay. Only a small amount of MTT formazan product can be detected in the growth medium of wells in which cells have been exposed to MTT. Comparative chemosensitivity data for EMT6 mouse tumour cells show good agreement between results obtained using the MTT assay and results based on total cell count after a fixed period of growth.


Br. J. Cancer (1987), 56, 279–285 © The Macmillan Press Ltd., 1987

### A study of some variables in a tetrazolium dye (MTT) based assay for cell growth and chemosensitivity

P.R. Twentyman & M. Luscombe

*MRC Clinical Oncology and Radiotherapeutics Unit, Hills Road, Cambridge, CB2 2QH, UK.*

**Summary** We have studied various factors involved in the optimal use of a tetrazolium (MTT) based colorimetric assay for cell growth and chemosensitivity. The assay is dependent on the ability of viable cells to metabolise a water-soluble tetrazolium salt into a water-insoluble formazan product. We have found that DMSO is the best solvent for dissolving the formazan product, especially where a significant amount of residual medium is left in the wells of the microtitre tray used for the assay. A reaction occurs between medium and a solution of MTT formazan in DMSO which changes the shape of the absorbance spectrum of the solution. The resulting optical density is not however greatly dependent upon the volume of added medium in the range 1–10 $\mu$l. Between 10 and 40 $\mu$l of added medium results in a gradually lower optical density than that produced by the smaller volumes. Above 40 $\mu$l, the optical density increases again due to turbidity as protein precipitation occurs. When cells are incubated with MTT, the resulting optical density of the formazan product is dependent upon both the concentration of MTT and the incubation time. The optical density is stable for several hours after solution of the formazan in DMSO. A linear relationship is seen between optical density and cell number for incubation times of 2, 4, 6 or 24 h with 20 $\mu$l of MTT (5 mg ml$^{-1}$) added to 200 $\mu$l medium. We have adopted 4 h as the standard incubation time for the assay. Only a small amount of MTT formazan product can be detected in the growth medium of wells in which cells have been exposed to MTT.

Comparative chemosensitivity data for EMT6 mouse tumour cells show good agreement between results obtained using the MTT assay and results based on total cell count after a fixed period of growth.

There are many applications in cancer research of assays which quantitate numbers of viable cells present following therapeutic procedures. Traditional assays have involved either counting total viable cells (using haemocytometer chambers or electronic particle counters) or colony counting. The need to process large numbers of samples has led to attempts to introduce assays which can be automated. Radionuclide incorporation assays have been extensively studied and widely used but usually include steps which are relatively time-consuming. More recently an extremely rapid colorimetric assay was described by Mosmann (1983). This assay involves the ability of viable cells to convert a soluble tetrazolium salt, 3-4,5 dimethylthiazol-2,5 diphenyl tetrazolium bromide (MTT), into an insoluble formazan precipitate (Slater *et al.*, 1963). The purple-coloured formazan crystals may be dissolved in a variety of organic solvents and the optical density of the resulting solution measured on a multiwell spectrophotometer (ELISA plate reader). This method has been used in a number of laboratories and various modifications have been introduced (Alley *et al.*, 1986; Carmichael *et al.*, 1987; Cole, 1986; Denizot & Lang, 1986). We wished to use the assay in our laboratory to study the response of established cell lines to cytotoxic drugs and also the ability of various compounds to modulate resistance in chemo-resistant cell lines. In this paper we describe our studies designed to examine the effect of a number of variables upon the results obtained using the assay in this context.

### Materials and methods

### *Cells and medium*

The cells used in this study were of the EMT6/Ca/VJAC mouse tumour cell line. These grow attached to plastic with a doubling time during exponential growth of ~12 h. We used Eagles minimal essential medium with Earles salts and 20% new born calf serum (except when specifically varied as part of a series of experiments) (all Gibco Biocult Ltd).

Correspondence: P.R. Twentyman.
Received 16 February 1987; and in revised form, 21 April 1987.

Cells for experiments were taken from exponential phase cultures growing in 75 cm tissue culture flasks. Disaggregation was carried out using a 15 min incubation at 37°C with a 0.05% solution of trypsin in phosphate-buffered saline (PBS).

### *MTT assay*

The tetrazolium salt, 3-4,5 dimethylthiazol-2,5 diphenyl tetrazolium bromide (MTT) and the corresponding MTT formazan were both obtained from Sigma. Solvents used were DMSO, acidified isopropanol (BDH) and mineral oil (Sigma). Optical density measurements were carried out using either a scanning spectrophotometer (Beckman model 25) or a Titertek Multiskan plate reader (ELISA reader) with a fixed wavelength filter. At the time of these experiments, a 600 nm filter was used although a 550 nm filter has subsequently been obtained. Measurements of the optical density of formazan solutions on the spectrophotometer were carried out using glass cuvettes. All measurements in the plate reader were carried out with the samples contained in 96-well multiplates (Falcon Plastics).

To study the effect of small volumes of culture medium or PBS on the optical density of formazan solutions, groups of wells were filled with 200 $\mu$l volumes of a series of formazan solutions of various concentrations in DMSO. Various volumes of medium or PBS were then added to the wells. Alternatively, serial dilutions of medium or PBS in DMSO were prepared and 20 $\mu$l added to the wells. Plates were agitated on a shaker for 5 min and optical density was read.

In experiments to study the production of formazan by EMT6 cells, the cells were grown in 96 well plates in volumes of 200 $\mu$l of medium per well. MTT was dissolved in PBS, usually at a concentration of 5 mg ml$^{-1}$, sterilised by filtration, and a volume of 20 $\mu$l added to each well. After a further period of incubation (usually 4–6 h), the medium was aspirated from the wells as completely as possible without disturbing the formazan crystals and cells on the plastic surface. A 200 $\mu$l volume of solvent (usually DMSO) was added to each well, the plates agitated on a plate shaker for 5 min and the optical density then read. These variables were specifically altered in some experiments.

The effect of changing the serum content of the medium present during the time following MTT addition was studied by removing the growth medium from wells, rinsing once with 200 $\mu$l of the test medium, and then again adding 200 $\mu$l of the test medium to each well. The plates were then returned to the incubator to allow return to 37°C before addition to MTT. In other experiments we looked at the effect of rinsing the cell monolayer following exposure to MTT but before addition of DMSO. We also examined the medium from wells in which MTT conversion had occurred to measure the amount of formazan product present in the medium rather than trapped in the cells.

In all these experiments, either 3 or 4 replicate wells were used to determine each point.

*Chemosensitivity assays*

A direct comparison was made of the effects of adriamycin (ADM, Pharmitalia), vincristine (VCR, Eli Lilley) or melphalan (MEL, Chester Beatty Research Institute) upon the growth of EMT6 cells as assayed either by cell counting or by the MTT assay. ADM and VCR were dissolved in distilled water whilst MEL was dissolved in acidified ethanol. Appropriate solvent controls were included in all experiments. For cell counts, groups of triplicate 6 cm plastic tissue culture petri dishes were set up, each dish containing $4 \times 10^4$ cells in 5 ml of medium. For MTT assay, wells were set up, also in triplicate, each well containing $10^3$ cells in 200 $\mu$l medium. For continuous drug exposure experiments, drugs were added immediately after the dishes/wells were set up and left in throughout the incubation period. In other experiments where a 1 h drug exposure was used, this was carried out in suspension, followed by a triple rinse, immediately before the dishes/wells were set up. The dishes/wells were incubated for 72 h. At the end of this time, cells in dishes were trypsinised from the surface and counted using a Coulter ZB2 electronic particle counter. MTT (20 $\mu$l of 5 mg ml$^{-1}$) was added to each well of the multiwell plates and the plates incubated for 4 h. The medium was then removed, 200 $\mu$l of DMSO added to each well and the plates agitated for 5 min. The optical density was then read at 600 nm on the plate reader.

### Results

The reproducibility of the MTT assay was extremely good. Where no cells were involved, triplicate wells generally gave values within 2% of the mean (see for example Table I). Where cells were involved, the errors were a little larger (see Figure 5) but were in many cases smaller than the symbols used in the figures. Generally, therefore, error bars are not shown in individual figures. Each figure or table represents the values obtained in a single experiment (except where stated) but all experiments were carried out at least twice to ensure reproducibility.

**Table I** Effect of medium on the optical density of formazan solutions. Various volumes of medium were added to 200 $\mu$l samples of a 30 $\mu$g ml$^{-1}$ solution of MTT formazan in DMSO

| *Volume added* ($\mu$l) | *Optical density* |
|---|---|
| 0 | 0.407 (0.002) |
| 10 | 0.899 (0.009) |
| 20 | 0.771 (0.000) |
| 30 | 0.705 (0.009) |
| 40 | 0.675 (0.014) |
| 60 | 0.786 (0.015) |
| 80 | 0.914 (0.018) |
| 100 | 1.135 (0.009) |

Values are means of 3 wells and figures in parentheses are standard deviations.

*Solvents*

Following incubation of a fixed number of cells per well with MTT for 4 h and the aspiration of the bulk of the medium we used various solvents to dissolve the formazan crystals. We found that both mineral oil and acid isopropanol were slow to dissolve the crystals and that solubilisation was still incomplete after 5 min agitation on a plate shaker. The process could be completed by pipetting the contents of each well but this was obviously time consuming. Furthermore, we found that mineral oil was particularly poor at dissolving the crystals if a significant amount of residual medium was present in the wells. As we also wished to use this methodology for non-adherent cell lines (where residual medium is unavoidable) this is a clear disadvantage. Also we found that acid isopropanol caused precipitation of protein present in any residual medium and hence a high background of optical density. (This was, however, much less of a problem if foetal calf serum rather than new born calf serum were used.) Because of our desire to adopt a solvent system with universal applicability we therefore decided to use DMSO. This dissolved the formazan crystals extremely rapidly without excessive agitation. Its ability to dissolve various types of plastic should however be borne in mind when selecting dispensing apparatus.

*Optical properties*

The absorbance spectrum of an MTT formazan solution in DMSO was determined on the spectrophotometer and is shown in Figure 1a. Also shown in Figure 1a is the spectrum of the same solution of formazan DMSO following the addition of 0.5% v/v of medium (see below). Increasing the volume of added medium did not bring about any significant further change in the shape of the spectrum. Linearity between optical density and concentration of formazan was demonstrated using both the spectrophotometer (550 and 600 nm) and the plate reader (600 nm) (Figure 1b). At the time these studies were carried out we only had a 600 nm filter for the plate reader. Subsequent tests however have shown that the response curves for the plate reader at 550 and 600 nm are parallel, the optical density being ~1.7 times as high at 550 nm as at 600 nm.

*Effect of culture medium in formazan solution*

In order to study the effect of any variation in the volume of residual medium left in wells before addition of DMSO to formazan precipitate, a series of experiments was carried out in which various volumes of medium were added to formazan solutions of different concentration in DMSO. The results are shown in Figure 2. It may be seen that a considerable change in optical density was brought about by the addition of volumes of medium as small as 2 $\mu$l (Figure 2a). Using a formazan concentration of 30 $\mu$g ml$^{-1}$, we studied a complete range of added volumes of medium. The data from one such experiment are shown in Table I. It is seen that the addition of 10 $\mu$l causes a large increase in optical density. Increasing the added medium volume to 40 $\mu$l causes a progressively smaller increase than does 10 $\mu$l, but beyond 40 $\mu$l, the optical density again begins to increase. We then went on to study the effect of adding 20 $\mu$l of successive 1:1 dilutions of medium in DMSO to 200 $\mu$l of formazan solution. The results of a typical experiment are shown in Figure 3. The MTT formazan solution used in this experiment was 30 $\mu$g ml$^{-1}$. Optical density was measured immediately after addition of the medium and 5 min agitation. Also shown in Figure 3 are the results of a similar experiment using dilutions of PBS in DMSO instead of medium in DMSO. The shape of these curves were very reproducible in repeat experiments (including the small peak

**Figure 1** (a) Absorbance spectrum of MTT formazan solution in DMSO as measured on the spectrophotometer. Dotted line – without addition of medium. Solid line – following addition of 0.5% v/v Eagles medium; (b) relationship between optical density and concentration of MTT formazan solution in DMSO: ●, measured on spectrophotometer at 550 nm; ▲, measured on spectrophotometer at 600 nm; △, measured on plate reader at 600 nm. The original MTT formazan solution in DMSO was $0.25\,mg\,ml^{-1}$. Each serial dilution represents a reduction by a factor of 2 in concentration.

**Figure 2** Effect of added medium on optical density of formazan solutions measured on the plate reader at 600 nm. Various volumes of medium were added to 200 μl samples of MTT formazan solutions in DMSO. The formazan solutions of different concentration were prepared as described in the legend to **Figure 1**. Two separate experiments are shown in (a) and (b): ●, control (no medium added); ▲, 2 μl added; ■, 10 μl added; △, 20 μl added; □, 40 μl added; ○, 80 μl added.

in the curve seen at a 6-fold dilution of PBS in DMSO). It is clear that 20 μl of a 6-fold dilution of medium in DMSO, added to 200 μl of formazan solution can cause a considerable change in optical density. This is equivalent to a volume of ~0.3 μl medium. Studies using the spectrophotometer showed that the changes in optical density caused by medium addition corresponded to a shift in the shape of the absorbance spectrum of the formazan in DMSO from that shown as the dotted line in Figure 1a to that shown by the solid line. We were also able to show that the major factor causing the spectral change was the sodium bicarbonate content of the medium.

These results taken together indicate that volumes of medium of ~1 μl to 10 μl cause an approximately equal increase in optical density of the formazan solution. From 10 μl to 40 μl, the optical density is decreased, whilst above 40 μl it again increases. This latter increase is due to protein precipitation which occurs when very large volumes of medium are added. The initial changes in optical density for added volumes of 10–40 μl do not, however, depend upon the percentage of serum present in the medium (Table II). Addition of 20 μl of a $10\,mg\,l^{-1}$ solution of phenol red (i.e. the concentration present in Eagles medium) in DMSO to 200 μl of formazan/DMSO solution did not cause a significant change in optical density.

A rise in temperature was observed in tubes in which 10% of medium or PBS was added to a solution of formazan in DMSO, presumably as a result of an exothermic chemical reaction. This raised the question of whether the changes in optical density brought about by such addition were stable with time. We therefore measured the optical density in wells at various times after the addition of medium or PBS. Results are shown in Figure 4. It is seen that no changes occurred over a period of 6 h in the increased optical density measured following addition of medium, but that the increased optical density following PBS addition fell again

**Figure 3** Effect of added medium or PBS on optical density of formazan solutions measured on plate reader at 600 nm. Serial dilutions of medium (●) or PBS (▲) in DMSO were prepared, and 20 $\mu$l samples of these were added to 200 $\mu$l volumes of MTT formazan solution in DMSO (30 $\mu$g ml$^{-1}$). Plates were agitated for 5 min and then optical density read immediately. Point A represents formazan solution with nothing added. Point B represents formazan solution with 20 $\mu$l DMSO added. Zero serial dilution means medium or PBS alone. Each serial dilution represents a 2-fold reduction in the concentration of medium or PBS in DMSO.

**Figure 4** Effect of added medium or PBS on optical density of formazan solutions read on plate reader at 600 nm. Wells containing 200 $\mu$l of MTT formazan solution in DMSO (16 $\mu$g ml$^{-1}$) were prepared and 20 $\mu$l volumes of medium (●) or PBS (▲) were added. The optical density was then determined at various times afterwards with the plates stored on the bench at room temperature. Control wells (○) contained formazan solution in DMSO without any additions.

**Table II** Effect of medium on the optical density of formazan solutions. Various volumes of media with different serum contents were added to 200 $\mu$l samples of a 30 $\mu$g ml$^{-1}$ solution of MTT formazan in DMSO

| Volume added ($\mu$l) | % serum in medium added | | | |
|---|---|---|---|---|
| | 0 | 5 | 10 | 20 |
| 0 | 0.52 | | | |
| 10 | 1.23 | 1.22 | 1.22 | 1.17 |
| 20 | 1.13 | 1.06 | 1.05 | 1.03 |
| 30 | 0.97 | 0.93 | 0.91 | 0.89 |
| 40 | 0.82 | 0.80 | 0.81 | 0.80 |

Readings of optical density taken immediately after addition of medium and 5 min agitation.

over a period of 1 h. At a higher concentration of formazan, an optical density of 1.13 was increased to 1.48 immediately after addition of 10% PBS but fell to 1.20 over the subsequent hour.

*Time course of formazan development*

In order to study the time course of formazan development a series of experiments was carried out in which multi-well plates were set up with either $5 \times 10^3$ or $2 \times 10^4$ EMT6 cells/well. Eighteen hours later, MTT solution was added to the wells in varying amounts and concentrations. After different incubation times, the medium was aspirated from the wells and the formazan precipitate dissolved in DMSO. The plates were then agitated for 5 min, and the optical density read. The results are shown in Figure 5. It may be seen that the shape of the curves are very dependent upon both cell number and amount of MTT, ranging from clearly concave downwards to clearly concave upwards over the period 0–6 h. We also examined the relationship between optical density and number of cells per well after various times of incubation with 20 $\mu$l 5 mg ml$^{-1}$ MTT added to each well. The results are shown in Figure 6. It is seen that

**Figure 5** Optical density resulting from dissolving formazan product in DMSO following incubation of cells with MTT solution of various concentrations for various lengths of time. (a) $5 \times 10^3$ cells/well plated for 16 h before MTT addition; (b) $2 \times 10^4$ cells/well: ■, 20 $\mu$l/well of 1.25 mg ml$^{-1}$ MTT; ▲, 10 $\mu$l/well of 5 mg ml$^{-1}$ MTT; ●, 20 $\mu$l/well of 5 mg ml$^{-1}$ MTT. Error bars in (b) show ±2 standard errors of the mean of 4 wells/point. No error bars are shown when these are smaller than the symbol.

**Figure 6** Optical density resulting from dissolving formazan product in DMSO following incubation of different numbers of cells per well with 20 $\mu$l 5 mg ml$^{-1}$ MTT solution per well for various lengths of time. Initial cell concentration was $3.2 \times 10^5$ per well, each serial dilution represents a 2-fold reduction in number of cells per well: ▲, 2 h incubation; ●, 4 h incubation; ■, 6 h incubation; ○, 24 h incubation.

all the curves are linear up to an optical density of ~0.9. We decided to adopt standard conditions where an optical density approaching this value of 0.9 was attained with the cell number approaching the end of exponential growth. This was found to be the case for 4 h incubation with 20 $\mu$l MTT solution (5 mg ml$^{-1}$) added to 200 $\mu$l medium per well.

*Serum concentration*

As we wished to use the MTT assay for various cell lines which grow in medium with different percentages of added serum, we examined the effect of changing the medium present in wells containing EMT6 cells immediately before addition of the MTT. We also tried rinsing the wells either once or three times with serum-free medium before addition of DMSO. The results are shown in Figure 7. It is clear that formazan production from MTT is greatest at lower serum concentrations. Although rinsing did appear to remove this effect, the sensitivity of the assay was reduced presumably due to loss of formazan during the rinsing procedure. An exact repeat of this experiment gave essentially identical results.

*Distribution of formazan product*

Because of the above evidence that formazan could be lost during rinsing, possibly because of solubility in medium, we examined the distribution of product between cells and medium after MTT exposure. The medium was removed from identical cell-containing wells either immediately after, or 4 h after MTT addition. Medium from wells to which MTT had not been added was used as control. Samples of

**Figure 7** Optical density resulting from dissolving formazan product in DMSO following incubation of $2 \times 10^4$ cells per well with 20 $\mu$l of 5 mg ml$^{-1}$ MTT in medium containing different percentages of serum: ●, without rinsing; ▲, one rinse following MTT incubation period; ■, three rinses following MTT incubation period.

the various media were added to 200 $\mu$l DMSO in new wells and the optical density read. Results are shown in Figure 8. It may be seen that 20 $\mu$l residual MTT formazan contributes 0.02 in optical density (i.e. 0.2 for 200 $\mu$l), whereas the cells in this experiment produced an optical density of 0.84. It would therefore appear that product loss of formazan into the medium is significant but not great enough to represent a major problem in the assay.

**Figure 8** Optical density of 200 $\mu$l samples of DMSO following addition of samples of medium from wells in which incubation of cells with MTT has occurred. The plate reader was blanked (i.e. optical density = zero) for DMSO alone: ■, medium from wells without MTT; ▲, medium from wells with MTT added immediately before medium removed; ●, medium from well with MTT added 4 h before medium removed.

*Storage of MTT solution*

Four experiments were set up in which MTT solutions which had been prepared at various times prior to use and stored in the refrigerator were added to identical groups of cell-containing wells. The results (not shown) demonstrated that formazan production from the various MTT solutions was not reduced by storage of solutions for at least 6 weeks.

*Chemosensitivity testing*

The chemosensitivity of EMT6 cells to either 1 h or continuous exposure to ADM, VCR or MEL was determined using both final cell count and MTT assays. All experiments were carried out in duplicate. A set of results for ADM are shown in Figure 9, and the results of all the experiments are given as $ID_{50}$s in Table III. The $ID_{50}$ is defined as the drug dose required to reduce the final cell number or the optical density to 50% of the control value (i.e. without drug). It may be seen that there is generally very good agreement between the two assays. Although differences in $ID_{50}$ are seen in individual experiments these are not consistently in the direction of any one assay. EMT6 cells are very resistant to short exposures to VCR (Kwok & Twentyman, 1985) and this reflected in the results of the 1 h experiments. For the other 8 experiments shown the ratio of $ID_{50}$s (cell count: MTT) is 1.3 (s.d. = 0.3). It is clear from these data that the MTT assay provides essentially equivalent values for chemosensitivity as those provided by counting total cells at the end of a fixed culture period.

**Figure 9** Response of EMT6 cells to adriamycin either for continuous (a) or 1 h (b) exposure. Solid symbols – total cell count assay; Open symbols – MTT assay. Each point represents the mean value of 3 separate dishes (●) or wells (○) and the error bars in (a) show ±2 standard errors of the mean.

**Table III** Chemosensitivity of EMT6 cells assessed by cell count or by MTT assay

| *Drug* | *Exposure* | *ID50*[a] (*μg ml*$^{-1}$) | |
|---|---|---|---|
| | | *Cell count* | *MTT* |
| ADM | 1 h | 0.64 | 0.57 |
| | 1 h | 0.39 | 0.35 |
| | cont. | 0.059 | 0.045 |
| | cont. | 0.055 | 0.038 |
| VCR | 1 h | >8.0 | >8.0[b] |
| | 1 h | >4.0 | 1.7 |
| | cont. | 0.030 | 0.019 |
| MEL | 1 h | 4.4 | 2.8 |
| | cont. | 1.4 | 1.0 |
| | cont. | 0.9 | 1.3 |

[a]$ID_{50}$ = dose of drug required to reduce final cell number or optical density in MTT assay to 50% of control (see **Materials and methods**). [b]EMT6 cells are very resistant to a 1 h exposure to VCR. These figures are taken from curves which are extremely shallow and hence give very variable values of $ID_{50}$.

### Discussion

There is no doubt that the MTT assay has great potential as a rapid method of screening for drug responsiveness of cell lines. A number of different variations of the assay conditions have however been described in the literature and it remains unclear which provide for optimal speed and accuracy.

In the original study by Mosmann (1983) cells were grown and MTT was added in a 100 μl volume of medium. Acidified isopropanol was then added and the content of the wells 'mixed thoroughly' to dissolve the formazan crystals. It was considered that the presence of phenol red in the medium was not a major problem as this was turned yellow by the acid in the solvent, thereby contributing little to the optical density at the wavelength measured. The study by Cole (1986) followed the Mosmann method closely, except that cells were grown in 200 μl medium of which 100 μl was removed before MTT addition. The Mosmann method was, however, adapted by Denizot and Lang (1986) in a number of ways. Firstly, the medium in each well was replaced with serum-free medium immediately before MTT addition in order to circumvent the problem of protein precipitation. Secondly, phenol red was omitted from this serum-free medium and the isopropanol solvent was not acidified. This was because of worries regarding a shift in the formazan absorbance spectrum brought about by the presence of the acid. Thirdly, the MTT-containing medium was removed from the wells by 'inverting, flicking and blotting' the tray before addition of the propanol solvent.

Alternate solvents were studied by Alley *et al.* (1986) and by Carmichael *et al.* (1987). The former workers preferred DMSO to isopropanol on the grounds that DMSO provided better solubilisation of formazan as well as giving more stable and greater readings of optical density. Carmichael *et al.* (1987) followed Alley *et al.* (1986) in choosing DMSO for non-adherent cell lines but also found mineral oil satisfactory when almost complete removal of medium could be achieved before solvent addition.

Our own experience, especially with regard to the choice of a universally applicable solvent system, supports the preference of Alley *et al.* (1986) for DMSO. Many of our experiments use non-adherent lines of human small cell lung cancer and we are also working with human leukaemia specimens directly from patients. Even following centrifugation of plates (5 min at 200 *g*) it is impossible to remove all of the medium without disturbing and aspirating some of the formazan crystals. In general 10–20 μl of medium remain per well. We found that this residual medium greatly impeded solubilisation of formazan in either mineral oil or acidified isopropanol. This could be overcome by pipetting the contents of each well but this is a time-consuming and inconvenient step. Furthermore, precipitation of protein remained a considerable problem with isopropanol, especially in lines which grow in medium with high concentrations (up to 20%) of new-born calf serum. With DMSO as solvent, solubilisation of formazan is rapidly complete, even with up to 40 μl medium left per well. Furthermore, precipitation of protein is not a problem with up to this volume of medium containing 20% new-born calf serum. It is clear, however, that a chemical reaction takes place between medium and formazan in DMSO. Although the resultant optical density is dependent upon volume of medium, the variation over a medium volume from 1–20 μl is small. The reaction between

DMSO and medium considerably changes the absorbance spectrum of formazan in DMSO due to the sodium bicarbonate content of the medium. However, phenol red at $10\,mg\,ml^{-1}$ does not change the optical density of formazan in DMSO.

The time course of formazan development depends upon both cell density and amount of MTT added. However, at $5\,mg\,ml^{-1}$, a linear relationship between optical density and cell number is seen at both 2 h and 4 h. This is unlikely to be true for all concentrations of MTT and conversion times etc. Following solubilisation of MTT formazan in DMSO, the optical density is stable for several hours but NOT for as long as 24 h.

For a given cell concentration per well, formazan production is greatest in serum-free medium and decreases with increasing serum concentration. This fact may be utilisable in order to optimise formazan production by cell lines where production is normally low. It is also clear that although solubility of formazan in medium is low, it is by no means negligible. This makes rinsing of the contents of wells following conversion of MTT an undesirable procedure.

In this study, we have compared the MTT assay with total viable cell count as indicators of chemotherapy response. A satisfactory degree of agreement was seen between the results obtained using the two assays. It must be remembered that neither of these assays is equivalent to a clonogenic assay for a number of reasons. The clonogenic assay, whilst determining the proportion of cells with intact reproductive integrity, takes no account of reduced growth rate induced by a drug (which would show as a reduced mean size of colonies). Neither does it take into account any period of division delay which may be induced. The short and medium term response of tumours *in vivo* to chemotherapy may be as dependent upon these latter factors as upon cell kill (Twentyman, 1985). Hence a total cell count (or MTT) assay can add valuable and distinct information to that provided by clonogenic assay and may be seen as complementary.

We believe that the MTT assay using DMSO as a solvent is widely applicable for the purpose of determining cytotoxic drug sensitivity of cell lines and offers considerable advantages in terms of speed over other existing assays.

*Note added in proof*: The optical density of a chemical solution of MTT formazan in DMSO remains stable for several hours following addition of small volumes of medium (**Figure 4**). However, when DMSO is added to a small volume of medium containing unconverted MTT (even in the absence of cells), production of formazan proceeds to a significant extent over the next few hours. It is, therefore, important, when using DMSO as a solvent in the MTT assay, to read the plates as soon as possible after DMSO addition.

We thank Dr Paul Workman for helpful discussion of the data regarding changes in absorbance spectra of formazan/DMSO.

### References

ALLEY, M.C., SCUDIERO, D.A., MONKS, A., CZERWINSKI, M.J., SHOEMAKER, R.H. & BOYD, M.R. (1986). Validation of an automated microculture tetrazolium assay (MTA) to assess cell growth and drug sensitivity of human tumor cell lines. *Proc. Amer. Assoc. Cancer Res.*, p. 389 (*abstract*).

CARMICHAEL, J., DE GRAFF, W.G., GAZDAR, A.F., MINNA, J.D. & MITCHELL, J.B. (1987). Evaluation of a Tetrazolium-based semiautomated colorimetric assay: Assessment of chemosensitivity testing. *Cancer Res.*, **47**, 936.

COLE, S.P.C. (1986). Rapid chemosensitivity testing of human lung tumour cells using the MTT assay. *Cancer Chemother. Pharmacol.*, **17**, 259.

DENIZOT, F. & LANG, R. (1986). Rapid colorimetric assay for cell growth and survival. Modifications to the tetrazolium dye procedure giving improved sensitivity and reliability. *J. Immunol. Methods*, **89**, 271.

KWOK, T.T. & TWENTYMAN, P.R. (1985). The response to cytotoxic drugs of EMT6 cells treated either as intact or disaggregated spheroids, *Br. J. Cancer*, **51**, 211.

MOSSMANN, T. (1983). Rapid colorimetric assay for cellular growth and survival: Application to proliferation and cytotoxicity assays. *J. Immunol. Methods*, **65**, 55.

SLATER, T.F., SAWYER, B. & STRAULI, U. (1963). Studies on succinate-tetrazolium reductase systems. III. Points of coupling of four different tetrazolium salts. *Biochim. Biophys. Acta.*, **77**, 383.

TWENTYMAN, P.R. (1985). Predictive chemosensitivity testing. *Br. J. Cancer*, **51**, 295 (*editorial*).